# Household Tobacco Expenditure and Child Health Outcomes: Causal Evidence from a Transitional Economy

**DOI:** 10.3390/healthcare13243312

**Published:** 2025-12-17

**Authors:** Kim-Anh Tran, Mai-Trang Le, Yung-Fu Huang, Manh-Hoang Do

**Affiliations:** 1 Department of Business Administration, Chaoyang University of Technology, Taichung 413310, Taiwan; trankimanh@tmu.edu.vn; 2 Department of Economics, Thuongmai University, Hanoi 10000, Vietnam; lmtrang2000@tmu.edu.vn; 3 Department of Marketing and Logistics Management, Chaoyang University of Technology, Taichung 413310, Taiwan; t1995034@o365.cyut.edu.tw

**Keywords:** household tobacco expenditure, children’s health, economic, Vietnam

## Abstract

**Background/Objectives**: The relationship between household tobacco expenditure and child health has attracted considerable attention from both academic and policy communities, as tobacco expenditure can influence children’s health, nutrition, and overall well-being in multiple ways, particularly in rural and low-income settings. This study examines the causal impact of household tobacco expenditure on child health outcomes in a transitional economy. **Methods**: Using nationally representative microdata from the most recent Household Living Standards Survey, the authors employ Ordinary Least Squares (OLS), Random Effects (RE), and Instrumental Variable (IV) estimations to identify the effects of tobacco spending on children’s healthcare utilization and health status. **Results**: The results consistently show that higher household tobacco expenditure significantly increases the likelihood of hospitalization among Vietnamese children, with the effects being most pronounced for those under six years of age. Moreover, the authors uncover substantial heterogeneity across gender, maternal age at childbirth, and regional contexts, highlighting persistent socioeconomic inequalities in health outcomes. **Conclusions**: This study provides compelling evidence of the adverse effects of household tobacco expenditure on children’s health in Vietnam. Theoretically, the study contributes to the literature on the economics of health and intra-household resource allocation by providing micro-level causal evidence from a transitional setting. From a policy perspective, the findings underscore the need for targeted fiscal and public health interventions to mitigate tobacco-related welfare losses and to promote equitable access to healthcare among vulnerable populations.

## 1. Introduction

Tobacco use constitutes a substantial global health burden and remains one of the foremost causes of preventable morbidity and mortality worldwide. Estimates from the World Health Organization (WHO) indicate that tobacco use accounts for more than eight million deaths annually, of which over 1.3 million are attributable to exposure to secondhand smoke [1]. The detrimental effects of tobacco extend beyond users themselves, disproportionately affecting non-smoking individuals; particularly children, who face both immediate health risks from exposure to secondhand smoke and indirect harm through constrained household resource allocation [2,3].

Prenatal and postnatal exposure to secondhand smoke has been strongly linked to various health complications in children, including respiratory infections, low birth weight, stunted growth, and impaired cognitive development [4,5]. From an economic perspective, household spending on tobacco generates a cascade of indirect effects that exacerbate health inequities, particularly in resource-limited settings [6,7,8]. In low- and middle-income countries (LMICs), tobacco-related expenditures often divert resources from essential needs such as food, education, and healthcare. This financial trade-off disproportionately impacts marginalized populations, particularly women and children, undermining household stability and long-term well-being [9,10].

In Vietnam, household tobacco expenditure remains prevalent at alarmingly high rates, posing significant risks to child health and household economic stability [11,12]. Findings from the Vietnam Household Living Standards Survey (VHLSS) indicate that tobacco use disproportionately affects low-income households, reinforcing cycles of poverty and hindering developmental outcomes [11,12]. Moreover, tobacco expenditure diverts financial resources from essential needs, reducing expenditure on education and healthcare. This reallocation of funds can have detrimental effects on children’s development and overall well-being [12]. Similarly, M. N. Nguyen et al. [11] quantified the impoverishing effects of tobacco use and estimated that, in 2018, tobacco-related expenditures pushed approximately 305,090 individuals in Vietnam into poverty, with children accounting for a substantial share of those affected.

While existing literature highlights the multifaceted impacts of tobacco expenditure on household economics and adult health, critical gaps remain in understanding its intergenerational effects. Notably, evidence linking household tobacco expenditure to specific child health outcomes, particularly in emerging economies, is limited. Most studies have examined overall household resource allocation without considering age-specific vulnerabilities or regional disparities. Therefore, more granular analyses are needed to address these gaps and inform targeted policy interventions, particularly in Vietnam [12].

This study contributes to the literature by providing updated empirical evidence on the direct and indirect effects of household tobacco expenditure on child health outcomes in a developing country. Using data from the VHLSS, it analyzes the likelihood that Vietnamese children require healthcare, the frequency of hospital visits, and total healthcare expenditures. By examining age-specific and regional differences, this research offers nuanced insights into the broader socioeconomic dynamics shaping child health in LMICs. Aiming to address these gaps, this study poses the following research questions:

Q1. How does household tobacco expenditure affect the frequency of children’s hospital visits and total healthcare expenses across different age groups?

Q2. How does parental age at childbirth and children’s gender influence the relationship between household tobacco expenditure and children’s health outcomes across different demographic subgroups?

Q3. What is the role of regional and household-level factors in explaining variations in children’s health outcomes in the context of household tobacco expenditure?

By addressing these questions, our study aims to provide a nuanced understanding of the impact of household tobacco expenditure on children’s health in Vietnam. It enhances knowledge of this critical public health issue and informs policies to reduce tobacco-related health risks. Additionally, it highlights the economic burden on vulnerable families, offering insights to promote healthier and more equitable outcomes for children.

## 2. Literature Review

The relationship between household tobacco uses and child health has garnered significant academic and policy interest due to the multifaceted ways tobacco use affects children’s health, nutrition, and overall well-being [4,5,10]. Drawing on the determinants of child survival framework proposed by Mosley and Chen [13,14], this study examines the direct and indirect pathways through which household tobacco use impacts children, with a particular focus on developing economies.

### 2.1. Direct Health Impacts of Tobacco Use

Household tobacco use has direct implications for child health, primarily through secondhand smoke (SHS) exposure. SHS, a combination of mainstream and side-stream smoke, contains over 7000 toxic chemicals, many of which are known carcinogens and irritants [15,16,17]. Extensive research has demonstrated that prolonged SHS exposure exacerbates chronic health conditions in children, particularly during critical growth periods [4,10]. Evidence indicates that such exposure contributes to recurrent respiratory infections and impaired growth [5,9].

Additionally, Talukder et al. [18] found that children in smoking households in Albania faced significantly higher risks of malnutrition and stunting compared to those in non-smoking households. Studies from Southeast Asia further highlight that parental smoking increases the likelihood of respiratory infections, low birth weight, and infant mortality. For example, Andriani et al. [4] reported that parental smoking in Indonesia, Cambodia, and Laos was associated with higher under-five mortality rates, with maternal smoking exerting a particularly strong effect. Moreover, Astuti et al. [9] found that children exposed to cigarette smoke for more than three hours daily had a tenfold increased risk of stunting in Indonesia. Similarly, Cao et al. [5] demonstrated that household SHS exposure significantly reduced the height of school-aged children in China, underscoring a dose–response relationship between exposure duration and growth deficits.

Parental smoking is significantly associated with increased SHS exposure among school-aged children (12–15 years) [19]. Particularly in Vietnamese households where one or both parents smoke [20]. Additionally, Rang et al. [21] investigated the relationship between SHS exposure during pregnancy and preterm birth, finding that women who experienced preterm deliveries had significantly higher rates of SHS exposure compared to those with full-term pregnancies. Prenatal exposure to maternal smoking further exacerbates health risks. Studies have linked maternal smoking during pregnancy to low birth weight, preterm birth, and an increased risk of obesity in early childhood. Mittal [10] reported that higher incidences of low birth weight, preterm deliveries, and congenital anomalies among infants born to smoking mothers. Similarly, Cajachagua-Torres et al. [22] identified associations between maternal smoking and adverse cardiovascular and metabolic outcomes in children, including elevated body mass index (BMI) and increased fat-mass ratios. Postnatal exposure to tobacco smoke also poses significant health risks. Children exposed to SHS after birth are more likely to develop asthma, otitis media, and experience sudden infant death syndrome (SIDS) [23]. Furthermore, maternal smoking combined with cannabis use has been shown to exacerbate risks of obesity and altered hypothalamic–pituitary–adrenal axis functioning in children [22,24]. Additionally, Bhandari et al. [25] documented significant associations between maternal tobacco use during lactation and adverse child nutritional outcomes, including stunting and wasting.

### 2.2. Economic and Nutritional Consequences

Beyond its direct health effects, household tobacco expenditure significantly impacts child health by diverting resources away from essential expenditures [26]. In Vietnam, tobacco-related spending reduces household allocations for food, healthcare, and education, particularly in rural and low-income settings [11]. Thus, Nguyen et al. [11] estimated that tobacco use pushed approximately 305,090 individuals, including children, into poverty, disproportionately affecting marginalized populations.

Similar patterns have been observed across LMICs, where tobacco expenditures compete with essential needs, exacerbating economic vulnerabilities. In Mexico, Macías Sánchez and García Gómez [26] found that tobacco expenditure led to reduced spending on food and healthcare, with low-income households bearing the greatest burden. Their study estimated that nearly one million individuals were pushed below the poverty line due to tobacco-related expenses. In Indonesia, Swarnata et al. [27] reported that households allocating 10.7% of their monthly budget to tobacco products experienced significant reductions in spending on nutritious foods, such as meat and vegetables, worsening nutritional deficiencies. Similarly, in Serbia, Vladisavljevic et al. [28] found that tobacco expenditures limited household spending on food, clothing, and healthcare, particularly among low-income families. Consequently, the financial strain of tobacco expenditure extends to child health and development. Wu et al. [29] found that in households where both male and female members smoked, children were less likely to receive complete or even partial basic vaccinations and were provided with fewer protein-rich foods. This phenomenon is attributed to the compounded economic burden of dual tobacco expenditure, which depletes limited household resources and diverts funds away from critical health and nutrition needs [29]. Hence, these findings underscore the broader economic consequences of tobacco expenditure, which significantly detract from investments in child health and well-being.

### 2.3. Gendered Dimensions of Tobacco Use

The gendered nature of tobacco use significantly shapes its impact on household dynamics. While men are typically the primary individuals who smoke, women and children disproportionately bear the burden of SHS exposure and its associated health risks. Structural inequalities further limit women’s ability to influence household resource allocation or smoking behaviors. Studies in South Asia and Sub-Saharan Africa have shown that women in smoking households are more likely to experience intimate partner violence, compounding adverse effects on children [10]. Research from Indonesia and Albania similarly indicates that paternal smoking increases the risk of malnutrition and stunting in children [9,18].

Maternal smoking during pregnancy has distinct biological and developmental consequences. A study in South Africa by Modjadji and Pitso [30] found that maternal tobacco and alcohol use during pregnancy was strongly associated with stunting and underweight status among children under 12 months old. This dual exposure to nicotine and alcohol not only disrupts prenatal development but also reduces the quality and quantity of breast milk, exacerbating nutritional deficits [30].

## 3. Data and Methodology

### 3.1. Data Collection

The dataset employed in this study is derived from the VHLSS conducted by the Vietnamese General Statistics Office (GSO) to inform policy-making and socio-economic development planning. The survey, on average, samples approximately 47,000 households annually, providing a representative cross-section of 64 provinces nationwide, encompassing both urban and rural areas [31]. The VHLSS is meticulously designed to collect data across various levels—individual, household, and commune; thereby offering comprehensive insights into living standards and the socio-economic factors that impact well-being.

This study uses recently available data from 2010 to 2018 to explicitly investigate household tobacco expenditure and its impact on children’s health in Vietnam. The analysis focuses on households with children under 18 years of age, encompassing the entire pediatric population as defined by international standards [32,33,34]. The dataset integrates two levels of information: individual-level data on children’s demographic and health characteristics, and household-level data on tobacco expenditure. Children’s health is measured using three self-reported indicators: reported health problems, frequency of health checks, and total healthcare expenditure. To examine age-specific vulnerabilities to tobacco exposure, the study analyzes three age-based subsamples, including children under 18, under 10, and under 6 years of age, with corresponding sample sizes varying by estimation model. Specifically, the binary outcome analysis (Probit estimation) includes 110,255, 54,342, and 31,856 observations, respectively, reflecting less stringent data completeness requirements for the dichotomous healthcare utilization variable. A detailed description of selected variables is presented in Table 1.

For the main analytical models (OLS, RE, and IV estimations), the study employs complete-case samples consisting of 91,445; 49,720; and 29,339 observations, respectively. Estimations of Equations (1)–(3) are performed on the complete and subsample data to assess how the relationship between household tobacco expenditure and child health varies across age groups (see Table A1, Appendix A).

### 3.2. Analysis Techniques

The study employs multiple complementary econometric techniques to analyze the relationship between household tobacco expenditure and children’s health outcomes. Instead of testing formal statistical moderation (via interaction terms) or mediation (via indirect-pathway analysis), our approach emphasizes documenting heterogeneous effects across demographic and geographic subgroups and examining the independent contributions of various household and individual-level factors.

Specifically, aiming to answer our research questions by:

1. Age-stratified analysis: By estimating all models separately for three age-based groups (children under 18, under 10, and under 6 years), we document how the relationship between tobacco expenditure and health outcomes varies across age groups, with the expectation that younger children are more vulnerable to household tobacco expenditure.
2. Covariate analysis: The study includes parental age, children’s gender, household income, household size, and regional indicators as control variables to examine their direct relationships with health outcomes while accounting for tobacco expenditure. This helps identify which factors independently affect children’s health in households that consume tobacco.
3. Descriptive heterogeneity analysis: By conducting separate estimations and comparing coefficient magnitudes across different subsamples, we examine how patterns vary by demographic factors (e.g., comparing effects between male and female children, or children born to younger versus older mothers) without formally testing interaction effects. This method offers evidence of how different factors relate to health outcomes in various contexts.

This analytical approach offers strong evidence on how household tobacco expenditure affects children’s health differently across various contexts and populations, guiding targeted policy measures for at-risk groups. The combination of multiple econometric techniques, including Probit estimation, Ordinary Least Squares (OLS), Random Effects (RE) estimation, and Instrumental Variable (IV) estimation, across age-stratified samples, provides a thorough analysis of the complex relationships between tobacco expenditure and children’s health outcomes, as well as robust results, and addresses inherent challenges in observational health economics research.

#### 3.2.1. Probit Estimation

For the binary outcome of healthcare service utilization, the Probit model is employed to examine the binary outcome of whether a child necessitates medical intervention due to health complications potentially influenced by household expenditure on tobacco. This methodological choice is consistent with the standard application of Probit models in analyzing binary-dependent variables. Greene (2018) asserts that “the probit model is utilized when the dependent variable is binary, assuming values of 0 and 1” [35]. In this framework, the Probit model estimates the conditional probability that a child will experience a health issue requiring medical attention, contingent upon household tobacco expenditure. For instance, in research investigating smoking behavior and healthcare access, Probit models have been applied to predict the likelihood of daily smoking and its subsequent health-related consequences [36].

First, Probit estimation is detailed in Equation (1) below.

(1) $${hcheckdum}_{ijt}=\beta_{0}+\beta_{1}{tobaccoexp}_{jt}+X_{ijt}+Z_{jt}+u_{ijt}$$

The variable *hcheckdum* measures the frequency with which a child seeks healthcare at hospitals. It is a binary variable, taking a value of 1 if child *i* in household *j* seeks hospital care each year and 0 otherwise. Tobacco expenditure *(tobaccoexp)* refers to the total annual household expenditure on tobacco, including spending during special holidays. While smoking adversely affects both individuals who smoke and those exposed to secondhand smoke, household tobacco expenditure is hypothesized to increase the likelihood of children requiring healthcare. In some cases, secondhand smoke exposure leads to more severe health consequences than direct smoking. This impact is expected to be particularly pronounced in younger children, who are more vulnerable to tobacco exposure [37,38].

The vector *Xijt* includes individual-level control variables: child’s age (age), mother’s age at childbirth *(motherage)*, father’s age at childbirth *(fatherage)*, and school admission status *(schooladm)*. Age and Gender: Younger children have developed immune systems and face both immediate health risks from secondhand smoke exposure and greater vulnerability to infections [4,5]. Research demonstrates that early childhood is a critical period for lung development and immune system maturation, making younger children especially susceptible to environmental exposures including tobacco smoke [37,38]. This study expects (age) to be negatively associated with healthcare utilization. We also include (gender) (Male = 1, Female = 0) to account for known biological vulnerabilities and observed gender disparities in health outcomes. *Motherage* is a dummy variable equal to 1 if the mother was 35 or older at the child’s birth. Research suggests that mothers over 35 are 3.6 times more likely to experience adverse fetal outcomes compared to younger mothers [39], so we expect *motherage* to be positively associated with children’s healthcare needs. Similarly, *fatherage* is a dummy variable equal to 1 if the father was 40 or older at the child’s birth. The initial hypothesis for a positive association with children’s healthcare needs is rooted in the medical literature linking advanced paternal age to potential negative biological risks at conception [40]. However, this expectation is balanced by the potential socioeconomic effect, as older fathers typically possess greater economic stability and resources, which could positively impact the child’s living environment and access to quality care. Without clear empirical consensus on which mechanism dominates, we treat this as an uncertain relationship to be determined empirically.

*Zijt* is a vector of household-level control variables, including total annual household income, household size, number of children, total annual working hours, and household location. High-income families tend to provide better care for their children due to greater financial resources. On the one hand, higher income enables better nutrition, sanitary living conditions, preventive healthcare access, and safer home environments, potentially reducing illness incidence. Studies demonstrate positive associations between household food expenditure (enabled by higher income) and child anthropometry, suggesting income supports health through improved nutrition [41,42]. On the other hand, economic theory and empirical evidence indicate that wealthier families face lower financial barriers to healthcare and are more willing to seek medical care even for minor ailments, potentially increasing healthcare utilization rates. Given these competing forces, the net effect on healthcare utilization cannot be predicted from theory alone and must be determined empirically. Household size *(hhsize)* represents the number of individuals living in the same household as the child. We hypothesize a negative association with healthcare utilization (i.e., a protective effect) because a larger household often implies increased caregiving, supervision, and social support from extended family members (grandparents, relatives), contributing to a healthier environment. The number of children in the household *(nofchildren)* is measured by children under 18 years of age in the household. We hypothesize a positive association with healthcare visits. This is driven by the principle of resource dilution: larger numbers of children lead to fewer financial and parental resources allocated per child, which may result in increased health risks and greater necessity for medical attention. Similarly, total household working hours *(hhworkinghour)* is anticipated to have a negative effect on children’s health, as parents with longer working hours may have less time to care for their children. Household income (*hhincome*) exhibits two competing effects on healthcare utilization. On one hand, higher income enables better nutrition, sanitary living conditions, and preventive healthcare, potentially reducing the incidence of illness. On the other hand, wealthier households tend to have both the capacity and the inclination to seek medical attention even for relatively minor conditions and may choose costlier treatment options. As a result, the overall effect of income on healthcare utilization is theoretically ambiguous and must be assessed empirically. Household location *(region)* accounts for demographic and cultural differences across geographic areas, which may influence family caregiving practices and healthcare access.

#### 3.2.2. Linear Models for Count and Continuous Outcomes

For the frequency of children’s healthcare visits and overall household healthcare expenditures, both OLS and RE estimation techniques are utilized to analyze household tobacco expenditure’s impact on the frequency of children’s healthcare visits and overall household healthcare expenditures. OLS is a foundational method for understanding these relationships within pooled cross-sectional data [43]. To mitigate the influence of time-variant factors and unobserved heterogeneity within the sub-samples, RE estimation is implemented, treating the dataset as panel data. As Hsiao [44] explains, panel data methodologies, including the RE model, facilitate the control of unobserved individual-specific effects that remain constant over time. The RE model assumes these unobserved individual effects are uncorrelated with explanatory variables, making it suitable for analyzing longitudinal data [44]. In the context of multilevel analyses concerning household expenditures, RE models have been applied to address unobserved heterogeneity across various levels, such as child gender, parental age categories, and regional location, thereby yielding more precise estimates of the impact of tobacco expenditure on healthcare expenditures [45]. Thus, the modified model is as follows:

(2) $${hcheck}_{ijt}=\;\beta_{0}+\;\beta_{1}{tobaccoexp}_{jt}+\;X_{ijt}+Z_{jt}+u_{ijt}$$

and

(3) $${hexpenditure}_{ijt}=\;\beta_{0}+\;\beta_{1}{tobaccoexp}_{jt}+\;X_{ijt}+Z_{jt}+u_{ijt}$$

In this case, *hcheck* measures the number of times a child requires hospital visits each year, while *hexpenditure* represents total household spending on children’s healthcare. Other variables remain consistent with the discussion in Equation (1). We apply OLS estimation to three subsamples of children under 18, 10, and 6 years of age. To account for variations over time, we also employ RE estimation, treating these subsamples as panel datasets. However, estimating Equations (2) and (3) may encounter endogeneity issues because the dependent and independent variables could be correlated with the error term. For instance, higher household income might increase both healthcare and tobacco expenditures, since greater financial resources enable households to cover more needs.

#### 3.2.3. IV Estimation

The study employs IV estimation to address potential endogeneity issues inherent in the analysis [46,47]. Endogeneity arises when explanatory variables, such as household tobacco expenditure, correlate with unobserved factors influencing the dependent variable, such as healthcare visits. By leveraging IV estimation, the study enhances the reliability of causal inferences, isolating the exogenous variation in tobacco expenditure through the instrumental variable.

Thus, this methodological approach effectively disentangles the causal effect of tobacco expenditure on children’s health from potential confounding factors. In previous research examining the causal relationship between mental health and substance use, IV estimation has been employed to address endogeneity concerns, using instruments such as the death of a close acquaintance to capture exogenous variations in mental health status [48]. To address this issue, the authors adopt IV estimation, with household income as an instrumental variable, across the three subsamples (children under 18, 10, and 6 years of age). The identification logic relies on the following reasoning:

Relevance condition: Household income is a strong predictor of tobacco expenditure. Higher-income households have greater discretionary spending capacity, enabling tobacco purchases without necessarily reducing essential expenditures like food and healthcare. Economic literature consistently documents positive income elasticities for tobacco spending, particularly in developing countries where tobacco is a normal good [12,26,27,28]. While the authors do not report formal first-stage F-statistics due to data limitations, the economic relationship between income and tobacco spending is well-established in prior literature.

Exclusion Restriction: We emphasize that the potential direct effects of household income on children’s health (e.g., better nutrition and food security [41,42] or access to private healthcare) are largely captured by including household income as an explicit control variable in our main OLS, RE, and IV models. The IV estimation therefore isolates the causal effect stemming purely from the exogenous variation in tobacco expenditure, mitigating the primary concern of unobserved income-related confounders. Notedly, our IV model is exactly identified (an instrumental variable for an endogenous variable). Therefore, we cannot perform restrictive over-identification tests (such as the Hansen J-test or Sargan test) to formally assess validity.

## 4. Results

The authors investigate the impact of tobacco expenditure on children’s health in three main aspects: the probability of children seeking healthcare at the hospital, how many times the children need health checks or treatment in the hospital, and households’ total healthcare expenditure on children’s health.

### 4.1. Probability of Healthcare Utilization: Probit Estimation Results

First, probit estimation is conducted on under-18, under-10, and under-6 groups to check for possibilities. The following estimations examine the relationship between tobacco expenditure and health checks and expenditures using several techniques, including OLS, RE, and IV estimations. Table 2 reports results from Probit estimation.

Our Probit estimation provides robust evidence of a positive relationship between household tobacco expenditure and the likelihood of children in the same household requiring hospital visits. Holding other factors constant, an additional one million VND (~40 USD) in annual household tobacco spending increases the probability of children under 18 needing hospital care by 0.1%. This effect is more pronounced for younger children, with increases of approximately 0.145% for those under 10 and 0.187% for those under six. As expected, younger children are the most vulnerable to household smoking.

Maternal age at childbirth also significantly influences children’s health outcomes. Our findings indicate that older maternal age at parturition increases a child’s vulnerability to the adverse effects of household smoking. In contrast, paternal age above 40 is associated with stronger health outcomes in children. Additionally, age is negatively correlated with the likelihood of hospital visits, with older children being 0.05% to 0.14% less likely to require medical care. Male children are found to have a higher risk of hospitalization compared to females. Household characteristics, including household size, income, and geographic location, also affect children’s health. While larger household size significantly decreases the probability of a child needing hospital care, higher household income is associated with a greater likelihood that children seek hospital care. It indicates that the nutritional benefits of higher income may be offset by increased healthcare utilization. Compared to the Red River Delta region, children in most other regions have a higher probability of requiring hospital care, except in the northern region.

### 4.2. Healthcare Frequency and Expenditure: OLS, RE, and IV Estimation Results

#### 4.2.1. Healthcare Visit Frequency Results

Across all three estimation methods, household tobacco expenditure is found to have a significant positive effect on the number of annual hospital visits. The OLS estimation results from Table 3 provide an initial benchmark, indicating that an additional million VND in annual household tobacco spending is associated with increases of 0.0926, 0.133, and 0.161 hospital visits for children under 18, under 10, and under 6, respectively (all significant at *p* < 0.01). Crucially, the preferred IV estimation results (see Table A3, Appendix A), which address potential endogeneity concerns, are consistently lower than the OLS estimates, specifically indicating increases of 0.081, 0.118, and 0.143 times for the same age groups. This systematic difference suggests a positive endogeneity bias in the OLS model, which is successfully corrected by the IV approach. Nevertheless, the progressive increase in effect size for younger age groups is highly consistent across both methods, strongly reinforcing the age-vulnerability gradient identified in the Probit analysis.

#### 4.2.2. Healthcare Expenditure Results

Healthcare expenditures follow a similar pattern, with tobacco expenditure generating substantial financial burdens on households. The OLS results presented in Table 3 show that a one million VND increase in tobacco spending is associated with additional annual healthcare costs of 118.0 thousand VND (for children under 18), 162.5 thousand VND (for children under 10), and 211.1 thousand VND (for children under 6). These initial OLS findings are remarkably consistent with the more robust IV estimation results (117.4, 160.7, and 211.7 thousand VND for the respective groups).

The minimal difference between the OLS and IV coefficients suggests that endogeneity bias is not a significant concern in the expenditure equation, which strengthens confidence in the magnitude of the estimated economic burden. This robust finding represents an approximate 12–21% increase in healthcare spending per unit increase in tobacco expenditure, definitively demonstrating the significant economic burden imposed by household smoking behavior, with the consequences clearly escalating for the youngest age cohort. Furthermore, this robust alignment strengthens our confidence in the validity of the chosen instrument (*hhincome*), providing retrospective evidence (evidence derived after the initial estimation) of its appropriateness despite the structural limitation of the exactly identified model and the current inability to report the First-Stage F-statistic.

While the effect is relatively modest for children under 18, it intensifies for younger age groups. The impact is more pronounced for children under ten and is highest for those under 6 years of age. Among children under six, household tobacco spending can nearly double both the number of hospital visits and total healthcare expenditures compared to the under-18 group. This finding is consistent across models, reinforcing the negative association between age and healthcare burden, suggesting that, holding other factors constant, younger children are more vulnerable to the detrimental effects of household tobacco expenditure.

#### 4.2.3. Demographic and Household Characteristics Effects

The following results examine the independent relationships between demographic and household characteristics and children’s health outcomes, while controlling tobacco expenditure. These findings help identify which factors are linked to increased healthcare use and spending, offering insights into the broader context in which tobacco expenditure impacts children’s health. By analyzing these relationships across three age-specific groups, we show how the influence of these factors varies at different stages of childhood.

Among personal characteristics, gender has the most significant impact on children’s healthcare, with male children seeking medical care more frequently than females (effects ranging from 1.82 to 7.70% higher probability of hospital visits). One possible explanation for this disparity is gender-based differences in parental attention and healthcare prioritization, particularly in rural and remote areas of Vietnam, where male children may receive preferential treatment. However, due to data limitations, we cannot draw definitive conclusions about this issue. Given its potential economic and social implications in addressing gender disparities in healthcare access, further in-depth surveys are needed to explore this phenomenon.

Parental age at childbirth also influences children’s health outcomes. Children born to mothers over 35 years of age are more likely to require hospital visits, whereas no significant association is found with fathers’ age. Additionally, school enrollment positively affects children’s health, as children who attend school tend to have fewer hospital visits and lower healthcare expenditures. This suggests that educational engagement may contribute to both physical and mental well-being. The finding is consistent with research conducted by Itriyeva [49], which further substantiates the role of educational settings in promoting children’s health outcome.

Household characteristics also play a role in children’s healthcare utilization. A higher number of children in the household is significantly associated with increased hospital visits across all age sub-samples. This pattern may reflect the principle of resource dilution; whereby financial and parental resources are spread more thinly as family size increases. Consequently, each child may face heightened health risks, leading to a greater need for medical care. Both the OLS and RE estimations (see Table A2, Appendix A) show a consistently positive association between household income and children’s healthcare needs. This finding suggests that while higher income likely confers nutritional benefits, its dominant effect is on increased healthcare seeking behavior. Regional disparities further influence healthcare patterns. Compared to other areas of Vietnam, children in the Red River Delta region (the baseline group) have fewer hospital visits. However, healthcare expenditures in this region are higher, likely due to increased living costs.

## 5. Discussions

This study underscores the significant adverse effects of household tobacco expenditure on children’s health, particularly evident in increased hospital visits, higher healthcare expenditures, and the heightened vulnerability of younger children. Unlike Trinh et al. [50], which examined household expenditure more broadly in relation to child health outcome, this study specifically isolates and examines tobacco-related spending as a focal determinant of children’s health in Vietnam. By isolating tobacco expenditure as the primary explanatory variable of interest—rather than examining household consumption patterns more broadly, it offers a more precise analysis of how specific expenditure behavior adversely influences children’s health outcomes, thereby advancing understanding beyond general patterns of household spending. This distinction underscores the critical role of household resource allocation in shaping health trajectories, highlighting the disproportionate burden associated with tobacco expenditure.

This study examines heterogeneous effects of tobacco expenditure across different demographic subgroups through age-stratified analysis and documents the independent associations of various covariates with children’s health outcomes. Specifically, we do not test statistical interactions between tobacco expenditure and other variables (moderation), nor do we explicitly model indirect pathways through which tobacco affects health (mediation). Instead, the age-stratified estimations reveal differential impacts across age groups. At the same time, the inclusion of demographic and household-level covariates allows us to identify factors independently associated with children’s health in the context of tobacco expenditure. This approach provides policy-relevant insights into which populations are most vulnerable and which contextual factors contribute to health disparities, even without formal moderation or mediation testing.

The findings from our Probit and IV estimations reveal a direct and statistically significant association between household tobacco expenditure and children’s healthcare needs, with younger age groups, particularly those under six years—being disproportionately affected. This result is robust across all econometric specifications (OLS, RE, and IV), thereby strengthening causal inference and reinforcing the conclusion that younger children are especially vulnerable to the detrimental effects of household tobacco expenditure. This heightened susceptibility aligns with biological and epidemiological evidence showing that early childhood is a critical period for lung development and immune system maturation [37,38]. Our results also corroborate earlier studies in Vietnam, such as Suzuki et al. [51], who found that SHS exposure significantly increased hospitalization rates for pneumonia among children under five years old, and Miyahara et al. [52], who reported that paternal smoking elevated the risk of hospitalization for lower respiratory tract infections in young children. By quantifying both the economic and health burden across different age groups, our analysis extends this literature and provides clear evidence to support targeted interventions in households with very young children.

Beyond the primary findings on tobacco expenditure, our analysis of control variables reveals several statistically significant results that warrant further discussion.

Firstly, this study identifies notable gender disparities in healthcare utilization, with male children experiencing higher rates of hospital visits than females. This pattern is consistent across all three age-stratified subsamples, with the gender difference being particularly pronounced among children under six years of age. While this aligns with Suzuki et al. [51], who observed similar patterns among children exposed to ETS, our age-stratified analysis extends this finding by demonstrating that gender disparities in healthcare utilization persist across different developmental stages. This study highlights the potential influence of sociocultural biases in healthcare access and resource allocation that favor male children; an issue not explicitly examined in previous research. In contexts where cultural norms may prioritize healthcare for boys, this could lead to more frequent medical visits even for minor ailments, thereby increasing the observed hospitalization rate. This phenomenon underscores the need for further qualitative investigation into intra-household decision-making processes and the gender-based allocation of healthcare resources.

Secondly, while Lam et al. [20] found that maternal education, particularly at the college level or higher, reduces children’s exposure to SHS, this study also introduces an additional dimension by examining maternal age as a factor that exacerbates children’s vulnerability to tobacco exposure. Thus, the Probit estimation shows that older maternal age (>35) is significantly associated with an increased likelihood that children will need hospital care. Conversely, the authors found that older paternal age (>40) is associated with stronger health outcomes in children, a result that, while seemingly counterintuitive, may be explained by older fathers’ greater economic stability and resources, which could positively impact the child’s living environment and access to quality care. Our findings enrich the discussion on parental characteristics as key determinants of child health outcomes.

Thirdly, our findings confirm significant regional disparities and household characteristics in healthcare utilization. Children outside the Red River Delta face greater healthcare needs despite similar or lower healthcare expenditures. This finding is consistent with Trinh et al. [50], who documented geographic disparities in child health outcomes across Vietnam. These results strongly suggest structural inequities in healthcare access and quality, with families in certain regions having to seek medical care more often due to a lack of preventative care or lower quality of local healthcare services. In addition, the consistently positive relationship between income and children’s healthcare utilization, suggests that in Vietnam, while low-income households may face greater health risks, high-income households report higher utilization due to their financial capacity and reduced barriers to entry in the healthcare system.

Finally, from an economic perspective, our findings strongly support the “crowding-out” hypothesis, wherein tobacco expenditure diverts scarce household resources away from essential goods and services, including nutritious food, education, and healthcare. This is particularly detrimental in low-income households, where financial constraints amplify the trade-offs between tobacco expenditure and basic needs. This aligns with Humphries et al. [42], who found a positive association between household food expenditure and child anthropometry, suggesting that reductions in food spending due to tobacco expenditure could negatively affect children’s nutritional status. Additionally, Humphries et al. [41] demonstrated that food insecurity was significantly associated with lower height-for-age (HAZ) and Body Mass Index Z-score (BMI-Z) among Vietnamese children, further emphasizing the economic and nutritional repercussions of tobacco expenditure.

By addressing the specific impact of household tobacco expenditure while incorporating regional and gender-based analyses, this study advances the understanding of child health outcomes in Vietnam. However, it also underscores the need for further research into sociocultural dynamics and structural inequalities in healthcare access. Additionally, future studies should examine the long-term effects of secondhand smoke exposure on children’s cognitive development and educational attainment, as well as the complex interactions between tobacco expenditure, nutrition, and food security.

## 6. Research Implications

This study contributes to the growing body of literature on household tobacco expenditure and child health, offering actionable insights to improve public health outcomes in Vietnam and similar settings.

First, the findings underscore the urgent need for targeted public health interventions and policy reforms in a developing country. Children, particularly those under six years of age, are especially vulnerable to household tobacco expenditure. Policy efforts should extend beyond reducing tobacco use to discouraging smoking within household environments where children are present. Additionally, the Vietnamese government should establish designated smoking areas in public spaces to reduce overall exposure to secondhand smoke, including for children. While existing programs primarily focus on minimizing the effects of smoking in public areas, limited attention has been given to mitigating smoking at home and its impact on household members, particularly children. Notably, Vietnamese households with individuals who smoke should be encouraged to establish smoke-free zones to protect children from secondhand smoke exposure. Evidence from the United States [2], Taiwan [7], and Spain [8] demonstrates that voluntary smoke-free home rules can effectively reduce children’s exposure and improve the health of other nonsmoking household members. More importantly, raising awareness among smokers about the harmful effects of tobacco use on their family members’ health and well-being is essential for motivating them to reduce their tobacco consumption and adopt healthier household practices.

Second, policymakers must address the observed regional and gender disparities. Strengthening healthcare infrastructure and improving the quality of preventive care access in underserved regions, particularly the Mekong Delta and Highland areas, could help reduce geographic inequities. Simultaneously, educational campaigns aimed at transforming sociocultural norms may lead to gender-based resource allocation within families, promoting equitable healthcare access for all children, regardless of gender. Healthcare programs should include specific measures to promote equal treatment and attention for both male and female children, particularly in rural and traditional communities. Furthermore, integrating anti-smoking messages into maternal and paternal health programs could help mitigate intergenerational risks associated with tobacco use, including prenatal care, pediatric visits, and immunization campaigns. This integration can maximize reach while leveraging existing healthcare infrastructure.

Finally, for low-income households, financial counseling and support mechanisms, such as conditional cash transfers linked to health check-ups and smoke-free homes, could help alleviate the crowding-out effect and encourage investments in child health and nutrition.

## 7. Conclusions

This study provides compelling evidence of the adverse effects of household tobacco expenditure on children’s health in Vietnam, utilizing data from the VHLSS between 2010 and 2018. By applying robust econometric techniques such as Probit, OLS, RE, and IV estimation, the study demonstrates that higher household tobacco expenditure is significantly associated with increased hospital visits and greater healthcare expenditures for children, with the youngest age groups being the most vulnerable.

Key household and demographic factors, including maternal age, household income, gender, and region, are independently associated with these health outcomes across age groups and settings. Notably, older maternal age at childbirth is linked to elevated health risks for children in smoking households. Additionally, our age-stratified analysis reveals that male children have a higher frequency of hospital visits than females across all age groups, with this pattern particularly pronounced among children under 6 years of age. This consistent gender disparity may reflect underlying cultural preferences in healthcare-seeking behavior or in resource allocation within households. Regional disparities are also evident, as children outside the Red River Delta experience greater healthcare needs despite similar or lower expenditures, indicating inequalities in healthcare access and quality. Household income is significantly linked to a higher frequency of healthcare utilization. This finding is less indicative of poorer health among affluent children and instead reflects the crucial role of financial resources in facilitating healthcare access and promoting proactive health-seeking behavior. The consistency of these patterns across our three age-stratified subsamples (under 18, under 10, and under 6 years) provides robust evidence that these factors play important roles in shaping children’s health outcomes in the context of household tobacco expenditure.

Beyond its direct health consequences, household tobacco expenditure has broader socio-economic implications, exacerbating health disparities among children. By diverting resources from essential needs such as food and education, tobacco expenditure further compounds the vulnerability of children in low-income households. These findings highlight the necessity of targeted policies to mitigate both the direct and indirect health impacts of tobacco expenditure and associated tobacco use, particularly for young children who remain disproportionately affected.

Although this study makes an essential contribution to the existing literature, particularly in the context of Vietnam, several potential sources of bias and limitations should be acknowledged to interpret the findings and inform future research correctly.

Our analytical approach focuses on documenting heterogeneous effects across demographic subgroups and examining the independent associations of various factors with children’s health outcomes. The authors do not employ formal statistical tests for moderation (interaction effects) or mediation (indirect pathways). Although this limits our ability to draw definitive conclusions about how specific factors statistically shape the tobacco–health relationship or the mechanisms through which tobacco affects health, it nonetheless provides valuable descriptive evidence of differential vulnerability patterns across populations. Future research could extend this work by explicitly testing interaction terms between tobacco expenditure and demographic characteristics or by applying structural equation modeling to investigate potential mediating pathways through which tobacco expenditure influences children’s health (e.g., via nutritional status or household resource allocation). Such analyses would require more detailed data on potential mediators and sufficiently large sample sizes to ensure reliable identification of interaction effects.

The VHLSS employs stratified random sampling across Vietnam’s 64 provinces, achieving an approximately 95% response rate, substantially reducing the risk of selection bias. Nevertheless, selection effects may still arise if households with severe tobacco-related health problems are disproportionately more likely to participate in health surveys, potentially leading to an overestimation of tobacco’s health impacts. While our large sample size and national representativeness help mitigate this concern, selection bias cannot be entirely excluded. Data on household tobacco expenditure is based on self-reported annual spending, which may be affected by under-reporting due to social desirability or recall difficulties. Such misreporting could lead to an underestimation of the actual effect of tobacco expenditure on health outcomes. Similarly, the dependent variables (e.g., hospital visits, healthcare expenditures) are also self-reported, which introduces additional risk of response bias. Households may misremember the number of visits or exact spending amounts. Although these limitations cannot be eliminated, future surveys could improve accuracy by collecting data more frequently or incorporating objective measures where feasible.

The available data captures only annual household spending on tobacco as a proxy for exposure, without specifying the number of cigarettes consumed. The dataset also lacks information on which household members smoke, whether smoking occurs indoors or outdoors, and other contextual factors, such as children’s medical history, specific respiratory conditions, ventilation systems, and alternative sources of indoor pollution, that may influence outcomes. The absence of such detail constrains the ability to identify a direct causal link between tobacco use and child health. Future studies should prioritize collecting more granular data to enable more precise analyses and strengthen the evidence base for targeted interventions. Finally, future research should investigate the long-term effects of household tobacco expenditure on children’s health and development, including chronic illness trajectories, cognitive outcomes, and educational attainment.

## Figures and Tables

**Table 1:** Variable description.

Variable	Description	Measurement	Expected Direction ^1^
Outcome Variables			
Health check	Children need to seek healthcare	1 = child visited hospital, 0 = no hospital visit	
Hospital visits	The number of hospitals visit in a year	Count variable (0–80 visits)	
Healthcare expenditure	Total expenditure on children’s healthcare per year	Vietnamese Dong (VND), continuous	
**Main Exposure**			
Tobacco expenditure (*tobaccoexp*)	Household total tobacco expenditure per year	Millions of VND, continuous	+
**Individual Characteristics**			
Age	Age of children	Years, continuous (0–17)	−
Gender	Gender of children	Male = 1, Female = 0	
School enrollment (school)	Whether or not a child goes to school (binary variable, =1 if yes and 0 otherwise)		−
Father age (*fatherage*)	Age of the father at the time the child was born (binary variable)	=1 if age of the father > 40, =0 otherwise	Uncertain
Mother age (*motherage*)	Age of the mother at the time the child was born (binary variable)	=1 if age of the mother > 35, =0 otherwise	+
**Household Characteristics**			
Region\urban\rural	Location of where a child lives		
Household income (*hhincome*)	Total household income per year	Millions of VND, continuous	Uncertain
Household working hours (*hhworkinghour)*	Total household working hours per year	Hours, continuous	+
Household size	Total number of people living in the same house as the child	Count, continuous	−
Number of children	Children under 18 years of age in household	Count, continuous	+

^1^ Expected direction indicates the hypothesized sign of the coefficient based on theoretical frameworks and prior empirical literature. Positive (+) means the variable is expected to increase healthcare utilization; Negative (−) means expected to decrease utilization; “Uncertain” indicates competing theoretical mechanisms where existing literature does not provide clear directional predictions.

**Table 2:** Probit estimation.

Variables	Group 1	Group 2	Group 3
	Age < 18	Age < 10	Age < 6
Tobacco expenditure	0.100 ***	0.145 ***	0.187 ***
	(0.0103)	(0.0139)	(0.0184)
Total household working hour	−4.77 × 10^−6^ ***	−1.31 × 10^−6^	−3.62 × 10^−6^
	(1.66 × 10^−6^)	(2.20 × 10^−6^)	(2.73 × 10^−6^)
Household income	0.000604 ***	0.000714 ***	0.000924 ***
	(6.46 × 10^−5^)	(8.54 × 10^−5^)	(0.000111)
Household size	−0.0654 ***	−0.0654 ***	−0.0595 ***
	(0.00396)	(0.00516)	(0.00647)
Number of children	0.0105 **	0.00174	−0.000346
	(0.00444)	(0.00569)	(0.00697)
Age	−0.0555 ***	−0.126 ***	−0.139 ***
	(0.00128)	(0.00352)	(0.00461)
Gender (male = 1)	0.0204 **	0.0223 **	0.0362 **
	(0.00829)	(0.0112)	(0.0143)
Father age (>40 = 1)	−0.0394 ***	−0.0509 ***	−0.0613 ***
	(0.0133)	(0.0172)	(0.0217)
Mother age (>35 = 1)	0.0298 **	0.0218 *	0.0181 *
	(0.0130)	(0.0171)	(0.0222)
School enrolment	−0.268 ***	0.0274	0.0956
	(0.0143)	(0.0201)	(0.290)
Region ^1^			
Northern area	−0.0628 ***	−0.114 ***	−0.156 ***
	(0.0136)	(0.0176)	(0.0220)
Central	0.169 ***	0.167 ***	0.144 ***
	(0.0132)	(0.0176)	(0.0223)
Highland	0.248 ***	0.255 ***	0.229 ***
	(0.0172)	(0.0235)	(0.0304)
Southern area	0.299 ***	0.333 ***	0.296 ***
	(0.0160)	(0.0219)	(0.0284)
Mekong river delta	0.465 ***	0.440 ***	0.333 ***
	(0.0138)	(0.0187)	(0.0240)
Constant	0.252 ***	0.463 ***	0.496 ***
	(0.0202)	(0.0281)	(0.0346)
Observations	110,255	54,342	31,856

Notes: Standard errors in parentheses (*** *p* < 0.01, ** *p* < 0.05, * *p* < 0.1). ^1^ Red River Delta is the reference group for the region.

**Table 3:** OLS estimation.

Variables	Hospital Visit			Healthcare Expenditure		
	(1)	(2)	(3)	(1)	(2)	(3)
	Age < 18	Age < 10	Age < 6	Age < 18	Age < 10	Age < 6
Tobacco expenditure	0.0926 ***	0.133 ***	0.161 ***	118.0 ***	162.5 **	211.1 *
	−0.0168	−0.0279	−0.04	−42.66	−76.77	−123.2
Household working hour	4.78 × 10^−6^ **	1.06 × 10^−5^ ***	1.24 × 10^−5^ ***	−0.00254	0.00372	0.00818
	−2.31 × 10^−6^	−3.39 × 10^−6^	−4.61 × 10^−6^	−0.00669	−0.011	−0.0179
Household income	0.000573 ***	0.000794 ***	0.000984 ***	0.681 ***	0.64	0.567
	−8.39 × 10^−5^	−0.000127	−0.000171	−0.254	−0.405	−0.667
Household size	−0.0778 ***	−0.0947 ***	−0.107 ***	−35.68 ***	−37.31 **	−24.69
	−0.00533	−0.00833	−0.011	−10.27	−14.92	−20.17
Number of children	0.0342 ***	0.0353 ***	0.0425 ***	4.659	−3.813	−24.64
	−0.00576	−0.00854	−0.011	−9.606	−15.4	−20.72
Age	−0.0564 ***	−0.127 ***	−0.136 ***	−4.716	−33.03 ***	−32.65 *
	−0.00175	−0.0054	−0.00773	−3.261	−10.57	−17.02
Father age	−0.0066	−0.0363	−0.0538	−34.9	−80.95	−198.6
	−0.02	−0.0296	−0.0447	−64.2	−107.4	−170
Mother age	0.0591 ***	0.0844 ***	0.0889 **	55.14	101.9	175.5
	−0.0194	−0.0296	−0.0453	−58.21	−97.27	−157.1
Gender	0.0161	0.0462 ***	0.0765 ***	65.98 ***	57.11	86.47
	−0.0105	−0.017	−0.0246	−22.27	−35.4	−54.9
School enrolment	−0.315 ***	−0.022	0.0212	−148.6 ***	−21.91	−31.92
	−0.0197	−0.03	−0.362	−37.61	−57.68	−130.8
Region						
Northern	−0.0836 ***	−0.160 ***	−0.257 ***	−89.47 **	−132.1 **	−154
	−0.0126	−0.0203	−0.0281	−39.46	−66.33	−103.1
Central	0.173 ***	0.204 ***	0.224 ***	−133.4 ***	−191.2 ***	−247.5 ***
	−0.0143	−0.0226	−0.0328	−28.61	−49.23	−73.3
Highland	0.232 ***	0.282 ***	0.292 ***	1.136	−59.08	−76.68
	−0.019	−0.0327	−0.0473	−53.26	−80.42	−120.6
Southern	0.399 ***	0.532 ***	0.618 ***	−88.43 ***	−154.4 ***	−191.7 ***
	−0.0235	−0.0394	−0.0584	−29.41	−40.82	−55.37
Mekong river delta	0.850 ***	1.049 ***	1.052 ***	−141.4 ***	−222.4 ***	−319.4 ***
	−0.0226	−0.0361	−0.0494	−36.28	−38.23	−50
Year dummies	Yes	Yes	Yes	Yes	Yes	Yes
Constant	1.589 ***	1.807 ***	1.834 ***	503.4 ***	615.0 ***	575.2 ***
	−0.0337	−0.0512	−0.07	−59.77	−106.7	−160.9
Observations	91,445	49,720	29,339	91,445	49,720	29,339
R-squared	0.098	0.088	0.071	0.002	0.002	0.002

Notes: Standard errors in parentheses are bootstrapped with 1000 replications (*** *p* < 0.01, ** *p* < 0.05, * *p* < 0.1). Red River Delta is the baseline group for region.

## Data Availability

The original contributions presented in this study are included in the article. Further inquiries can be directed to the corresponding author.

## Abbreviations

The following abbreviations are used in this manuscript:

LMICs	Low- and middle-income countries
OLS	Ordinary Least Squares
IV	Instrumental Variable
SHS	Secondhand Smoke
RE	Random Effects
VHLSS	Vietnam Household Living Standards Survey
WHO	World Health Organization (WHO)

## Appendix A

**Table A1:** Data summary.

Variable	Age < 18					Age < 10					Age < 6				
	Obs	Mean	Std. Dev.	Min	Max	Obs	Mean	Std. Dev.	Min	Max	Obs	Mean	Std. Dev.	Min	Max
Hospital visit	91,445	0.773263	1.735277	0	80	49,720	1.066714	1.999244	0	80	29,339	1.32029	2.195031	0	80
Healthcare expenditure	91,445	269.7032	3592.465	0	650,000	49,720	327.9013	4089.043	0	650,000	29,339	401.4099	5039.494	0	650,000
Tobacco expenditure	91,445	0.33298	0.408771	0	19.9	49,720	0.334446	0.414209	0	19.9	29,339	0.337574	0.414835	0	19.9
Household income	91,445	44.4428	75.72167	0	1788	49,720	48.44685	80.02874	0	1320	29,339	50.8505	81.65254	0	1068
Household working hour	91,445	3446.599	3042.926	0	28,842	49,720	3544.874	3188.181	0	28,842	29,339	3706.574	3342.982	0	28,842
Number of children	91,445	2.613363	1.248647	1	8	49,720	2.650362	1.255074	1	8	29,339	2.707182	1.289207	1	8
Age	91,445	8.689628	5.069782	0	17	49,720	4.674075	2.744727	0	9	29,339	2.719384	1.593782	0	5
Father age (=1)	18,124	0.416021	0.4929	0	1	10,021	0.509956	0.499906	0	1	5792	0.5779	0.493903	0	1
Mother age (=1)	44,656	0.488337	0.499867	0	1	29,542	0.594167	0.491057	0	1	19,546	0.666212	0.471573	0	1
Gender															
Female	91,445	0.479305	0.499574	0	1	49,720	0.47669	0.499461	0	1	29,339	0.476056	0.499435	0	1
Male	91,445	0.520696	0.499574	0	1	49,720	0.523311	0.499461	0	1	29,339	0.523944	0.499435	0	1
School enrolls															
0	91,445	0.358412	0.479537	0	1	49,720	0.65352	0.475853	0	1	29,339	0.999421	0.024065	0	1
1	91,445	0.641588	0.479537	0	1	49,720	0.34648	0.475853	0	1	29,339	0.000579	0.024065	0	1
Region															
Red river delta	91,445	0.199584	0.399690	0	1	49,720	0.206336	0.404678	0	1	29,339	0.211391	0.408302	0	1
Northern	91,445	0.22595	0.418209	0	1	49,720	0.243061	0.428936	0	1	29,339	0.247214	0.431399	0	1
Central	91,445	0.228214	0.419683	0	1	49,720	0.218262	0.413071	0	1	29,339	0.217254	0.412384	0	1
Highland	91,445	0.089431	0.285366	0	1	49,720	0.083568	0.276742	0	1	29,339	0.080882	0.272659	0	1
Southern	91,445	0.09585	0.294387	0	1	49,720	0.091673	0.288567	0	1	29,339	0.089028	0.284789	0	1
Mekong river delta	91,445	0.160971	0.367506	0	1	49,720	0.1571	0.363899	0	1	29,339	0.154232	0.361177	0	1

**Table A2:** RE estimation.

Variables	Hospital Visit			Healthcare Expenditure		
	(1)	(2)	(3)	(1)	(2)	(3)
	Age < 18	Age < 10	Age < 6	Age < 18	Age < 10	Age < 6
Tobacco expenditure	0.0831 ***	0.120 ***	0.145 ***	113.0 ***	158.2 **	206.5 *
	−0.0166	−0.0269	−0.0402	−42.6	−66.77	−112.7
Total household working hour	4.16 × 10^−6^ *	9.26 × 10^−6^ ***	1.14 × 10^−5^ **	−0.00297	0.00324	0.00655
	−2.38 × 10^−6^	−3.36 × 10^−6^	−4.55 × 10^−6^	−0.00724	−0.0111	−0.0185
Household income	0.000549 ***	0.000775 ***	0.000966 ***	0.677 **	0.631	0.61
	−8.87 × 10^−5^	−0.000132	−0.000187	−0.279	−0.425	−0.683
Household size	−0.0694 ***	−0.0868 ***	−0.101 ***	−32.78 ***	−34.42 **	−22.79
	−0.00586	−0.008	−0.0117	−11.06	−16.06	−20.83
Number or children	0.0251 ***	0.0274 ***	0.0380 ***	0.657	−6.587	−26.9
	−0.00635	−0.00895	−0.0121	−11.48	−16.11	−23.07
Age	−0.0577 ***	−0.117 ***	−0.117 ***	−6.304 **	−33.80 ***	−32.22 *
	−0.00176	−0.00515	−0.00814	−3.012	−10.28	−17.15
Father age	−0.0102	−0.0428	−0.0595	−36.95	−83.59	−199.6
	−0.0204	−0.0303	−0.0424	−65	−101.1	−170.1
Mother age	0.0646 ***	0.0895 ***	0.0969 **	54.24	100.3	175.2
	−0.0194	−0.0298	−0.045	−60.01	−91.97	−155.8
Gender	0.00803	0.0334 *	0.0695 ***	67.05 ***	60.88 *	88.53 *
	−0.011	−0.0172	−0.0242	−22.71	−33.61	−51.75
School enrollment	−0.240 ***	−0.0261	−0.103	−125.1 ***	−15.76	−31.41
	−0.026	−0.0241	−0.248	−45.49	−41.35	−104.9
Region						
Northern	−0.0831 ***	−0.163 ***	−0.257 ***	−81.72 **	−120.3 *	−145.6
	−0.0131	−0.021	−0.0291	−41.59	−64.71	−105.1
Central	0.170 ***	0.202 ***	0.220 ***	−126.9 ***	−181.1 ***	−241.7 ***
	−0.0142	−0.0233	−0.0336	−30.7	−51.15	−74.13
Highland	0.233 ***	0.286 ***	0.296 ***	8.95	−48.44	−71.25
	−0.0196	−0.0332	−0.0478	−58.55	−83.36	−122.7
Southern	0.393 ***	0.528 ***	0.612 ***	−85.65 ***	−144.9 ***	−188.3 ***
	−0.0234	−0.0369	−0.059	−30.23	−39.79	−56
Mekong river delta	0.846 ***	1.041 ***	1.042 ***	−136.0 ***	−214.1 ***	−314.8 ***
	−0.0233	−0.0371	−0.0519	−35.87	−39.48	−49.22
Year dummies	Yes	Yes	Yes	Yes	Yes	Yes
Constant	1.545 ***	1.764 ***	1.774 ***	496.4 ***	603.5 ***	571.4 ***
	−0.0359	−0.0533	−0.0696	−62.18	−102.8	−159.6
Observations	91,445	49,720	29,339	91,445	49,720	29,339
Number of participants	89,612	48,837	28,910	89,612	48,837	28,910

Notes: Standard errors in parentheses are bootstrapped with 1000 replications (*** *p* < 0.01, ** *p* < 0.05, * *p* < 0.1). Red River Delta is the baseline group for region.

**Table A3:** IV estimation.

Variables	Hospital Visit			Healthcare Expenditure		
	(1)	(2)	(3)	(1)	(2)	(3)
	Age < 18	Age < 10	Age < 6	Age < 18	Age < 10	Age < 6
Tobacco expenditure	0.0812 ***	0.118 ***	0.143 ***	117.4 ***	160.7 ***	211.7 ***
	(0.0144)	(0.0223)	(0.0323)	(31.32)	(47.74)	(76.83)
Household total working hours	8.15 × 10^−6^ ***	1.58 × 10^−5^ ***	1.90 × 10^−5^ ***	0.00447	0.0104	0.0145
	(2.01 × 10^−6^)	(3.03 × 10^−6^)	(4.14 × 10^−6^)	(0.00438)	(0.00647)	(0.00983)
Number of children	−0.0262 ***	−0.0334 ***	−0.0326 ***	−25.71 **	−33.18 **	−44.44 *
	(0.00469)	(0.00728)	(0.0102)	(10.21)	(15.56)	(24.16)
Age	−0.0553 ***	−0.125 ***	−0.136 ***	−4.573	−32.39 ***	−32.74 *
	(0.00189)	(0.00541)	(0.00791)	(4.117)	(11.57)	(18.79)
Father age	−0.0558	−0.107	−0.141	−58.57	−110.4 **	−220.6 **
	(0.0176)	(0.0259)	(0.0366)	(38.22)	(55.38)	(86.82)
Mother age	0.0479 ***	0.0598 **	0.0536	50.66	92.36 *	167.4 *
	(0.0173)	(0.0262)	(0.0381)	(37.55)	(56.08)	(90.43)
Gender	0.0182 *	0.0476 ***	0.0770 ***	66.84 ***	57.58	86.42
	(0.0109)	(0.0172)	(0.0248)	(23.80)	(36.70)	(58.86)
School enrollment	−0.316 ***	−0.0304	0.0124	−148.5 ***	−25.62	−35.29
	(0.0198)	(0.0309)	(0.515)	(43.13)	(66.05)	(122.3)
Region						
Northern	−0.109 ***	−0.190 ***	−0.291 ***	−113.2 ***	−153.8 ***	−172.0 *
	(0.0169)	(0.0259)	(0.0371)	(36.81)	(55.42)	(88.10)
Central	0.163 ***	0.191 ***	0.208 ***	−144.6 ***	−202.0 ***	−257.3 ***
	(0.0169)	(0.0265)	(0.0380)	(36.66)	(56.60)	(90.25)
Highland	0.209 ***	0.257 ***	0.261 ***	−23.62	−81.24	−96.74
	(0.0222)	(0.0356)	(0.0517)	(48.34)	(75.95)	(122.9)
Southern	0.407 ***	0.548 ***	0.637 ***	−81.44 *	−146.0 **	−185.2
	(0.0215)	(0.0342)	(0.0497)	(46.86)	(73.07)	(118.0)
Mekong river delta	0.826 ***	1.018 ***	1.016 ***	−162.3 ***	−242.8 ***	−336.4 ***
	(0.0185)	(0.0291)	(0.0419)	(40.20)	(62.07)	(99.51)
Year dummies	Yes	Yes	Yes	Yes	Yes	Yes
Constant	1.386 ***	1.562 ***	1.567 ***	413.2 ***	520.0 ***	516.0 ***
	(0.0268)	(0.0432)	(0.0601)	(58.41)	(92.38)	(142.8)
Observations	91,445	49,720	29,339	91,445	49,720	29,339
R-squared	0.096	0.085	0.067	0.002	0.002	0.002

Note: Standard errors in parentheses (*** *p* < 0.01, ** *p* < 0.05, * *p* < 0.1). Household income is instrumental variable.

## References

[1] World Health Organization (WHO) (2023). WHO Report on the Global Tobacco Epidemic, 2023: Protect People from Tobacco Smoke.

[2] Tripathi O., Parada H., Shi Y., Matt G.E., Quintana P.J.E., Liles S., Bellettiere J. (2024). Perception of Harm Is Strongly Associated with Complete Ban on In-Home Cannabis Smoking: A Cross-Sectional Study. BMC Public Health.

[3] Asi E., Gozum S. (2025). Secondhand Smoke Exposure at Home Among Children Under the Age of Five: A Cross-Sectional Study. J. Child Fam. Stud..

[4] Andriani H., Putri S., Kosasih R.I., Kuo H.W. (2019). Parental Smoking and Under-Five Child Mortality in Southeast Asia: Evidence from Demographic and Health Surveys. Int. J. Environ. Res. Public Health.

[5] Cao S., Xie M., Jia C., Zhang Y., Gong J., Wang B., Qin N., Zhao L., Yu D., Duan X. (2022). Household Second-Hand Smoke Exposure and Stunted Growth among Chinese School-Age Children. Environ. Technol. Innov..

[6] Shehab K., Ziyab A.H. (2021). Beliefs of Parents in Kuwait about Thirdhand Smoke and Its Relation to Home Smoking Rules: A Cross-Sectional Study. Tob. Induc. Dis..

[7] Wang Y.T., Tsai Y.W., Tsai T.I., Chang P.Y. (2017). Children’s Exposure to Secondhand Smoke at Home before and after Smoke-Free Legislation in Taiwan. Tob. Control.

[8] Lidón-Moyano C., Martínez-Sánchez J.M., Fu M., Ballbè M., Martín-Sánchez J.C., Martínez C., Fernández E. (2017). Secondhand Smoke Risk Perception and Smoke-Free Rules in Homes: A Cross-Sectional Study in Barcelona (Spain). BMJ Open.

[9] Astuti D.D., Handayani T.W., Astuti D.P. (2020). Cigarette Smoke Exposure and Increased Risks of Stunting among Under-Five Children. Clin. Epidemiol. Glob. Health.

[10] Mittal S. (2019). Smoking and Tobacco Use: Ill Effects on Reproductive, Maternal, Newborn, Child Health, and Adolescent (RMNCHA) Program—A Review. Ann. Natl. Acad. Med. Sci..

[11] Nguyen M.N., Nguyen A.N., Bui H.T., Vu L.H. (2022). Impoverishing Effect of Tobacco Use in Vietnam. Tob. Control.

[12] Nguyen N.M., Nguyen A. (2020). Crowding-out Effect of Tobacco Expenditure in Vietnam. Tob. Control.

[13] Mosley W.H., Chen L.C. (1984). An Analytical Framework for the Study of Child Survival in Developing Countries. Child survival: Strategies for research. Popul. Dev. Rev..

[14] Samuel O., Zewotir T., North D. (2024). Application of Machine Learning Methods for Predicting Under-Five Mortality: Analysis of Nigerian Demographic Health Survey 2018 Dataset. BMC Med. Inform. Decis. Mak..

[15] Hackshaw A., Rodeck C., Boniface S. (2011). Maternal Smoking in Pregnancy and Birth Defects: A Systematic Review Based on 173 687 Malformed Cases and 11.7 Million Controls. Hum. Reprod. Update.

[16] Scherman A., Tolosa J.E., McEvoy C. (2018). Smoking Cessation in Pregnancy: A Continuing Challenge in the United States. Ther. Adv. Drug Saf..

[17] Dempsey D.A., Benowitz N.L. (2001). Risks and Benefits of Nicotine to Aid Smoking Cessation in Pregnancy. Drug Saf..

[18] Talukder A., Hasan M.M. (2022). Asikunnaby Assessing Association between Paternal Smoking Status and Child Malnutrition in Albania: An Application of Ordinal Regression Model. Hum. Nutr. Metab..

[19] Wang Q. (2021). Underweight, Overweight, and Tobacco Use among Adolescents Aged 12-15 Years: Evidence from 23 Low-Income and Middle-Income Countries. Tob. Induc. Dis..

[20] Lam N.T., Nga P.T.Q., Van Minh H., Giang K.B., Hai P.T., Huyen D.T., Linh N.T., Van D.K., Khue L.N. (2016). Trends in Second-Hand Tobacco Smoke Exposure Levels at Home among Viet Nam School Children Aged 13-15 and Associated Factors. Asian Pac. J. Cancer Prev..

[21] Rang N.N., Hien T.Q., Chanh T.Q., Thuyen T.K. (2020). Preterm Birth and Secondhand Smoking during Pregnancy: A Case-Control Study from Vietnam. PLoS ONE.

[22] Cajachagua-Torres K.N., El Marroun H., Reiss I.K.M., Santos S., Jaddoe V.W.V. (2022). Foetal Tobacco and Cannabis Exposure, Body Fat and Cardio-Metabolic Health in Childhood. Pediatr. Obes..

[23] Marbin J., Balk S.J., Gribben V., Groner J. (2021). Health Disparities in Tobacco Use and Exposure: A Structural Competency Approach. Pediatrics.

[24] Cajachagua-Torres K.N., Jaddoe V.W.V., de Rijke Y.B., van den Akker E.L.T., Reiss I.K.M., van Rossum E.F.C., El Marroun H. (2021). Parental Cannabis and Tobacco Use during Pregnancy and Childhood Hair Cortisol Concentrations. Drug Alcohol Depend..

[25] Bhandari Y., Das A., Aditi A., Kishore J., Goel S. (2024). Tobacco and Alcohol Use among Lactating Women and Its Association with Child Nutrition in India: Findings from National Family Health Survey 2019–2021. Public Health.

[26] Macías Sánchez A., García Gómez A. (2024). Crowding out and Impoverishing Effect of Tobacco in Mexico. Tob. Control.

[27] Swarnata A., Kamilah F.Z., Wisana I.D.G.K., Meilissa Y., Kusnadi G. (2024). Crowding-out Effect of Tobacco Consumption in Indonesia. Tob. Control.

[28] Vladisavljevic M., Zubović J., Jovanovic O., Aukic M. (2024). Crowding-out Effect of Tobacco Consumption in Serbia. Tob. Control.

[29] Wu D.C., Shannon G., Reynales-Shigematsu L.M., Saenz de Miera B., Llorente B., Jha P. (2021). Implications of Household Tobacco and Alcohol Use on Child Health and Women’s Welfare in Six Low and Middle-Income Countries: An Analysis from a Gender Perspective. Soc. Sci. Med..

[30] Modjadji P., Pitso M. (2021). Maternal Tobacco and Alcohol Use in Relation to Child Malnutrition in Gauteng, South Africa: A Retrospective Analysis. Children.

[31] GSO (2020). Results of the Vietnam Household Living Standards Survey 2018.

[32] Grigg J. (2009). Particulate Matter Exposure in Children: Relevance to Chronic Obstructive Pulmonary Disease. Proc. Am. Thorac. Soc..

[33] Campbell M.A., Winnall W.R., Ford C. (2021). 4.17 Health Effects of Secondhand Smoke for Infants and Children. Tobacco in Australia: Facts and Issues.

[34] World Health Organization (WHO) Adolescent Health. https://www.who.int/health-topics/adolescent-health?.

[35] Greene W. (2018). Econometric Analysis.

[36] Rezayatmand R., Groot W., Pavlova M. (2017). Smoking Behaviour and Health Care Costs Coverage: A European Cross-Country Comparison. Int. J. Health Econ. Manag..

[37] Flor L.S., Anderson J.A., Ahmad N., Aravkin A., Carr S., Dai X., Gil G.F., Hay S.I., Malloy M.J., McLaughlin S.A. (2024). Health Effects Associated with Exposure to Secondhand Smoke: A Burden of Proof Study. Nat. Med..

[38] Asfaw S.M., Vijayawada S.M., Sharifian Y., Choudhry F., Khattar P., Cavalie P.C., Malasevskaia I. (2024). Protecting Young Lives: A Systematic Review of the Impact of Secondhand Smoke Exposure and Legislative Measures on Children’s Health. Cureus.

[39] Damtew B.S., Hailu A.M., Fente B.M., Workneh T.W., Abdi H.B. (2024). Determinant of Adverse Early Neonatal Outcomes Following Emergency Cesarean Section in North West, Ethiopia: Institutionalbased Case-Control Study. BMC Pregnancy Childbirth.

[40] Denomme M.M., McCallie B.R., Haywood M.E., Parks J.C., Schoolcraft W.B., Katz-Jaffe M.G. (2024). Paternal Aging Impacts Expression and Epigenetic Markers as Early as the First Embryonic Tissue Lineage Differentiation. Hum. Genom..

[41] Humphries D.L., Dearden K.A., Crookston B.T., Fernald L.C., Stein A.D., Woldehanna T., Penny M.E., Behrman J.R., Georgiadis A., Cueto S. (2015). Cross-Sectional and Longitudinal Associations between Household Food Security and Child Anthropometry at Ages 5 and 8 Years in Ethiopia, India, Peru, and Vietnam. J. Nutr..

[42] Humphries D.L., Dearden K.A., Crookston B.T., Woldehanna T., Penny M.E., Behrman J.R. (2017). Household Food Group Expenditure Patterns Are Associated with Child Anthropometry at Ages 5, 8 and 12 Years in Ethiopia, India, Peru and Vietnam. Econ. Hum. Biol..

[43] Wooldridge M.J. (2010). Econometric Analysis of Cross Section and Panel Data.

[44] Hsiao C. (2022). Analysis of Panel Data.

[45] Do Y.K., Bautista M.A. (2015). Tobacco Use and Household Expenditures on Food, Education, and Healthcare in Low- and Middle-Income Countries: A Multilevel Analysis. BMC Public Health.

[46] Angrist J.D., Imbens G.W., Rubin D.B. (1996). Identification of Causal Effects Using Instrumental Variables. J. Am. Stat. Assoc..

[47] Saridakis G., Lai Y., Muñoz Torres R.I., Gourlay S. (2020). Exploring the Relationship between Job Satisfaction and Organizational Commitment: An Instrumental Variable Approach. Int. J. Hum. Resour. Manag..

[48] Mitrou F., Nguyen H.T., Le H.T., Zubrick S.R. (2024). The Causal Impact of Mental Health on Tobacco and Alcohol Consumption: An Instrumental Variables Approach. Empir. Econ..

[49] Itriyeva K. (2024). Improving Health Equity and Outcomes for Children and Adolescents: The Role of School-Based Health Centers (SBHCs). Curr. Probl. Pediatr. Adolesc. Health Care.

[50] Trinh T.A., Srivastava P., Brown S. (2022). Household Expenditure and Child Health in Vietnam: Analysis of Longitudinal Data. J. Demogr. Econ..

[51] Suzuki M., Thiem V.D., Yanai H., Matsubayashi T., Yoshida L.M., Tho L.H., Minh T.T., Anh D.D., Kilgore P.E., Ariyoshi K. (2009). Association of Environmental Tobacco Smoking Exposure with an Increased Risk of Hospital Admissions for Pneumonia in Children under 5 Years of Age in Vietnam. Thorax.

[52] Miyahara R., Takahashi K., Anh N.T.H., Thiem V.D., Suzuki M., Yoshino H., Tho L.H., Moriuchi H., Cox S.E., Yoshida L.M. (2017). Exposure to Paternal Tobacco Smoking Increased Child Hospitalization for Lower Respiratory Infections but Not for Other Diseases in Vietnam. Sci. Rep..

